# Modulation of prey capture kinematics in relation to prey distance helps predict success

**DOI:** 10.1242/jeb.247311

**Published:** 2024-06-12

**Authors:** Christopher E. Oufiero, Lohitashwa Garikipati, Elizabeth McMillan, Mary Katherine Sullivan, Ryan Turnbaugh

**Affiliations:** Department of Biological Sciences, Towson University, Towson, MD 21252, USA

**Keywords:** Predation success, Praying mantis, Behavior flexibility, ***Sphodromantis lineola***

## Abstract

Predators are not perfect, as some of their prey capture attempts result in failure. Successful attempts may be partly due to predators modulating their capture kinematics in relation to variation in the visual cues of the prey to increase the probability of success. In praying mantises, which have been suggested to possess stereoscopic vision, variation in prey distance has been shown to elicit variation in the probability of an attempt. However, it remains to be examined whether variation in prey distance results in mantises modulating their attempt to successfully capture prey. The goals of this study were to examine these relationships using the praying mantis system. Using 11 adult female *Sphodromantis lineola*, we recorded 192 prey capture attempts at 1000 Hz with two cameras to examine the 3D kinematics of successful and unsuccessful prey capture attempts. Using a combination of principal component analysis (PCA) and logistic regression, our results show that as prey distance increases, mantises adjust through greater and faster expansion of the forelegs and body (PC1), which significantly predicts capture success. However, PC1 only explains 22% of the variation in all prey capture attempts, suggesting that the other components may be related to additional aspects of the prey. Our results suggest that the distances at which mantises prefer to attempt to capture prey may be the result of their greater probability of successfully capturing the prey. These results highlight the range of motions mantises use when attempting to capture prey, suggesting flexibility in their prey capture attempts in relation to prey position.

## Teaser

**Summary:** Praying mantises modulate the kinematics of both forelegs in response to prey distance and angle, but only the changes in relation to prey distance predict prey capture success.

## INTRODUCTION

An inherent principle of predator–prey interactions is that in order for one to succeed the other must fail, resulting in predators that are not always successful in their attempts. The mechanics of prey capture have been examined in detail for many different types of organisms, almost always on successful attempts (Bub and Bowerman, 1979; Copeland and Carlson, 1979; Corrette, 1990; Deban, 1997; Ferry-Graham et al., 2001a,b; Flammang et al., 2009; Holzman and Wainwright, 2009; Longo et al., 2016; Nishikawa and Roth, 1991; Oufiero, 2022; Oufiero et al., 2012, 2016; Wainwright et al., 2001). This is in part due to the nature of the research, as one cannot examine the kinematic basis of prey capture if the predator does not capture the prey. Studies on the behavior and kinematics of prey capture have shown that these behaviors can exhibit variation in the kinematics both within and among species (Kane and Higham, 2011; Oufiero, 2022; Oufiero et al., 2012, 2016; Tran et al., 2010). However, prior studies on the kinematic basis of prey capture success may represent only a portion of actual attempts (Abrams, 1989; Vermeij, 1982). Some studies have investigated potential determinants or prey capture success, demonstrating that variation in the timing and positioning of the prey can contribute to a successful attempt (Higham et al., 2017; Maie et al., 2014; Milton and Bergmann, 2023; Peterson and McHenry, 2022). Although these prior studies have begun to show why a predator may fail, few have examined whether visual aspects of the prey, such as their distance from the predator, relate to the variation in movements to successfully capture prey. If predators are adjusting their prey capture strikes based upon variation in the visual stimulus to increase their chances of success, this would suggest they are modulating their prey capture mechanics and the response is flexible (Wainwright et al., 2008).

Flexibility in prey capture strikes is often the result of modulation of the movements in response to visual aspects of the prey (Deban, 1997; Ferry-Graham et al., 2001a,b; Lambert et al., 2011; Nemeth, 1997; Van Wassenbergh et al., 2006). Changes in the kinematics in response to visual cues to the prey suggest that predators are using visual cues to adjust their movements, likely to provide a greater chance for success. For example, within and among fish, the distance at which a predator initiates a strike has been shown to relate to various aspects of the prey capture strike, such as how much the mouth opens and the body speed used (Oufiero et al., 2012; Tran et al., 2010). For organisms with overlapping fields of view (i.e. stereoscopic vision), the distance between predator and prey has been shown to result in variation in their probability of initiating a prey capture attempt. Recent work in praying mantises (Mantodea) has demonstrated that they respond to various visual cues of prey, including prey distance (Nityananda et al., 2016a, 2019; Prete et al., 2013; Rossel, 1983, 1991). Using adult female *Sphodromantis lineola*, Nityananda et al. (2016a) demonstrated that restrained mantises have a preferred striking distances of 2.5 cm, as indicated by a greater probability of initiating a prey capture attempt at that distance compared with a few other prescribed distances. This preferred distance is likely related to the point of overlapping fields of view of their two main compound eyes, which varies ontogenetically and interspecifically (Kral and Poteser, 2009; Oufiero, 2020).

Although mantises have a preferred strike distance, they still vary in the distances at which they will initiate a prey capture attempt. For example, Nityananda et al. (2016a) also demonstrated that some mantises will attempt prey capture across a range of prey distances, depending on the absolute and angular size of the prey. Furthermore, mantises have neurons that preferentially respond to targets from 2.5 to 10 cm (Rosner et al., 2020). Variation in preferred distance to capture prey has also been demonstrated ontogenetically, matching predictions of variation in interocular distance variation, and has been shown to relate to variation in kinematics, such as the amount of body used during a prey capture attempt (Oufiero, 2022). However, it has yet to be demonstrated whether the variation in prey distance at which praying mantises attempt to capture prey is used to modulate their kinematics to successfully capture the prey. That is, is variation in prey distance causing a flexible response to increase the chances of prey capture?

The goals of this study were to: (1) build upon previous studies of successful prey capture attempts under semi-natural conditions by incorporating unsuccessful attempts, (2) determine the distances over which praying mantises attempted a prey capture event in unrestrained individuals under semi-natural conditions, (3) determine the relationship between prey distance with the kinematic and behavioral components of a prey capture attempt, and lastly (4) determine whether the relationships between prey distance and kinematics predict success in prey capture. We evaluated these goals using adult female *Sphodromantis lineola* praying mantises, a species that has been used for virtual prey experiments (Nityananda et al., 2016a). These goals were assessed with 3D kinematics from high-speed videos of successful and unsuccessful prey capture attempts in a semi-natural laboratory setting, where prey were tethered, but allowed to vary in their position during prey capture attempts.

## MATERIALS AND METHODS

Eleven adult female *Sphodromantis lineola* (Burmeister 1838) of similar size were used for this study. Mantis body length (as measured as the distance between pro- and meso-thorax legs, see below) averaged 2.16±0.132 cm (s.d.) with total foreleg lengths averaging 5.02±0.262 cm. Mantises were obtained from the hobby industry and raised in the lab from a juvenile stage on a diet of fruit flies (*Drosophila hydei*) and mealworms (*Tenebrio molitor*). Mantises were housed in 32 oz. (∼946 ml) plastic containers with ventilated lids, kept and filmed at an average of 24°C. Mantises were misted daily to provide proper hydration. Mantises were starved at least 24 h prior to filming prey capture events to ensure they were motivated to eat.

Prey capture attempts were filmed with two Edgertronic SC1 (Sanstreak Corporation, San Jose, CA, USA) high-speed cameras, synced within 1 µs, at 1000 Hz and a shutter speed of 50 µs. Mantises were removed from their housing container and placed on a custom filming stage (Fig. S1) in an upright position. The filming stage was illuminated with a Westcott SKYLUX 1000 W LED light and four CM Vision IR lights (850 nm); the IR lights provide illumination for the camera sensors filming at the higher frame rate, without adding additional visible light to the filming arena. Consistent prey (mealworms) were weighed prior to introducing them to the mantis on the filming stage, to obtain a measure of prey size. To simulate an evasive prey, the mealworm was manually moved in a random fashion (e.g. side to side, front to back and/or circular movement) in the field of view of the mantis to get the mantis to cue in on the prey and initiate a prey capture strike, similar to prior studies (Oufiero, 2022; Oufiero et al., 2016). The random motion of the prey ensured mantises were attempting a prey capture event at a range of distances to better simulate a semi-natural prey capture attempt. If the prey was too far, the mantis would not attempt to capture the prey, as it was likely out of range (Nityananda et al., 2016a,b). If the prey was caught, the mantis was allowed to consume the prey, then another prey was weighed and introduced. If it missed, defined as not grasping the prey and bringing it towards its mandibles, it was given 2–5 min until the prey was introduced again. This process was repeated several times per mantis until it was no longer interested in the prey, as evidenced by grooming behavior, not initiating a prey capture strike, or walking around on the stage. At this time, the trial for the individual ended for a particular day. Trials were recorded consecutively throughout and the trial number was used as a covariate in subsequent analyses. Each mantis was tested over several days.

A total of 192 prey capture attempts were analyzed among the 11 mantises, with 110 (57.29%) unsuccessful attempts and 82 (42.71%) successful attempts. Each prey capture attempt was digitized in DLTdv8 (Hedrick, 2008; McHenry and Hedrick, 2023), marking 10 points on both the lateral and dorsal view (20 points total) from a few frames prior to the first movement of the mantis until a few frames after the tibia had completed flexing (Fig. S2). The views were calibrated with a 3D Lego structure with known dimensions, visible in each view, that encompassed the space the mantises occupied during a prey capture attempt. After the views were calibrated and points tracked, the calibrated *x*,*y*,*z*-coordinates were exported to obtain the 3D kinematics of each attempt. Custom R script modified from two-dimensional videos was used to obtain a series of kinematics in the three-dimensional space (Oufiero, 2022; Oufiero et al., 2016). From each attempt, the predator–prey distance was taken as the distance between the eye (point 7) and the prey (point 6) at beginning of the strike, indicated by the first signs of predator movement. Similar to previous studies, prey angle was obtained as the angle of a line from point 1 (insertion of coxa on pro-thorax) through point 7 (eye) to point 6 (prey), and represents how high the prey is relative to the mantis's eye. The maximum angle of each foreleg joint was obtained as in previous studies (Oufiero, 2022; Oufiero et al., 2016). For the coxa, it was the angle at point 1, for the femur/trochanter, the angle at point 2, and for the tibia, the angle at point 3.

**Fig. 1.**
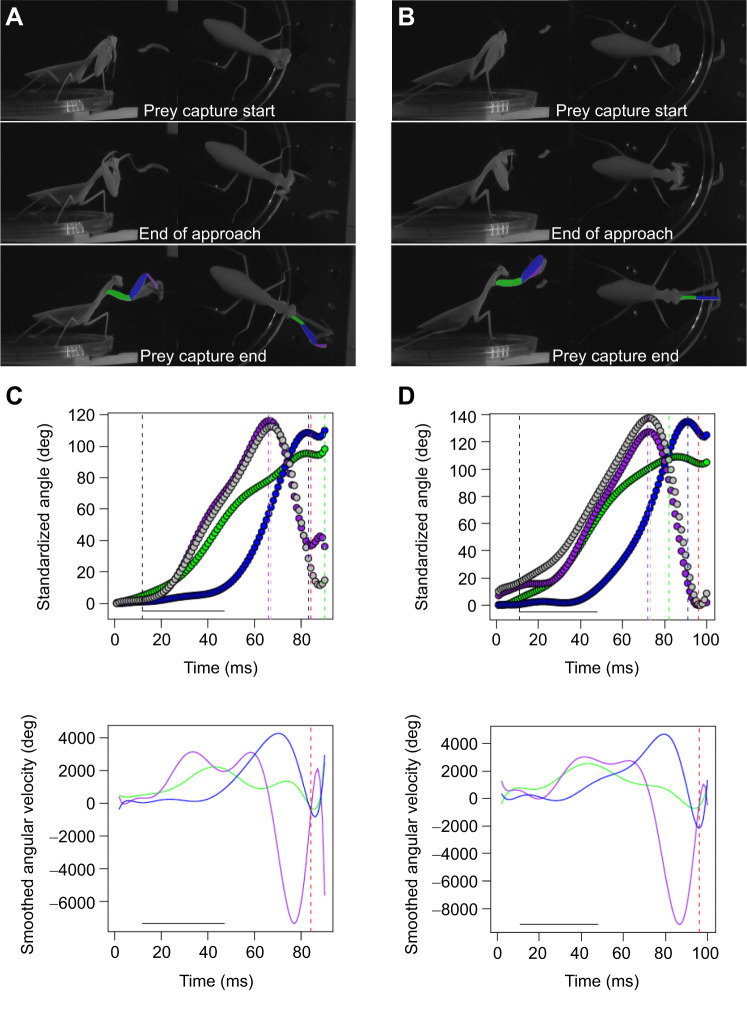
**Example prey capture strikes from the same individual on the same day.** Data are from (A,C) successful and (B,D) unsuccessful prey capture attempts. Video stills in A and B represent start of prey capture, which is also depicted as the start of the horizontal solid black line and the vertical black dashed line in C and D, end of the approach (end of the horizontal solid black line in C and D) and end of prey capture (dashed red line in C and D) from both synchronized cameras. (C,D) Example traces of standardized, smoothed angles (C) and angular velocities (D) of each joint [coxa in green, trochanter/femur in blue, front tibia in purple, back tibia in grey (standardized angles only)]. The traces allow for a comparison of the movement of each joint among prey capture events. Dashed colored lines represent peak values for each foreleg segment. Dashed red line represents the end of the prey capture strike, which was determined by the end of the peak using the findpeak function in R.

All angles were standardized based upon the starting angle and therefore represent the maximum angle that each segment opened, then smoothed using a 10th order polynomial following previous studies (deVries et al., 2012; Oufiero, 2022; Oufiero et al., 2016). The maximum coxa angle was taken as the 99% of the maximum angle, as it often continues to extend and never fully peaks (Fig. 1). For the femur/trochanter and tibia, the maximum angles were obtained using the findpeaks function in the pracma package in R. Because both segments open and then close, resulting in a peak in the angular kinematics (Fig. 1), the first major peak defines the point at which the tibia and femur are opened maximally. Therefore, the standardized, smoothed angle at that time point was taken as the maximum trochanter/femur and tibia angle. The resulting kinematics as shown in Fig. 1 were checked for all prey capture attempts to ensure there were no errors, which were corrected manually. The angular velocities were taken as the derivative of the standardized, smoothed angles against time and the maximums were taken as the maximum value for each. For the tibia, the lowest minimum was taken as it is fastest when flexing (resulting in negative values); therefore, we used the absolute values of the lowest minimum tibia velocities (Fig. 1). Body displacement was the movement of a point representing the mean between point 1 and 5 during the strike, which was smoothed with a 10th order polynomial. The linear velocity of body was the derivative of the smoothed body displacement against time. The time of the approach and sweep phases were defined following previous studies (Corrette, 1990; Oufiero, 2022; Oufiero et al., 2016), where the start of the approach was defined as the point when the coxa angle was at 5% of its maximum, as the coxa is often the first segment to extend, and ended when the trochanter/femur velocity was at 10% of its maximum, which captures when the trochanter/femur begins to rapidly extend. The end of the approach also signified the beginning of the sweep. As we were characterizing successful and failed prey capture attempts, we could not define the end of the sweep at prey capture as in previous studies (Corrette, 1990; Oufiero, 2022; Oufiero et al., 2016), as it requires the prey to pass into the space between the tibia and femur. Therefore, the end of the sweep was defined as the point when the tibia fully closed again, as defined from the findpeaks function, which provides the end of the peak.

Lastly, we obtained a new set of kinematic features for the praying mantis prey capture strike because we filmed in 3D: the lateral displacement of the coxa/trochanter joint, the femur/tibia joint and the tip of the tibia (Fig. 1). These were obtained by taking the maximum, smoothed displacement between each joint on the front and back foreleg. These traits represent how much the forelegs abduct laterally during the prey capture attempt, which may be important for a successful prey capture attempt.

For analyses, we first quantified the distribution of prey distances mantises used under our laboratory conditions. We compared the distances between successful and unsuccessful attempts using a one-way ANOVA with prey distance as the dependent variable and success as the independent variable. We repeated this for the angle of the prey at the start of the prey capture attempt, with prey angle as the dependent variable. To determine the relationship between prey distance and angle with the kinematic and behavioral components of a prey capture attempt we used principal component analysis (PCA) to reduce the axes of variation and examine correlation among traits. We condensed 15 kinematic variables (prey distance, prey angle, coxa, trochanter/femur and tibia maximum angles and angular velocities, approach time, sweep time, body displacement, body velocity, and lateral displacement of the coxa/trochanter, femur/tibia and tip of the tibia) to the main axes of variation using a PCA. Next, to determine which of the 15 PC axes we should examine, we used a broken stick model (Jackson, 1993; Oufiero et al., 2012), which examines which PC axes explain more variation than random. We used the bstick function in the vegan package for R for the broken stick model.

To determine whether the relationships between prey distance and kinematics predict success in prey capture, we used a logistic regression with success as the binary dependent trait and the significant PC axes as predictors using the glm function in R. We also explored the use of a mixed model logistic regression using the glmer function from the lme4 package, but the model was singular, suggesting the inclusion of individual mantis as a random effect did not have an effect. We also explored the effects of covariates such as prey mass, body length of the mantis obtained as the distance between points 1 (pro-thorax) and 5 (meso-thorax) legs and trial; however, none were significant and thus they were not included in the final model (*P*>0.05).

## RESULTS

**Fig. 2.**
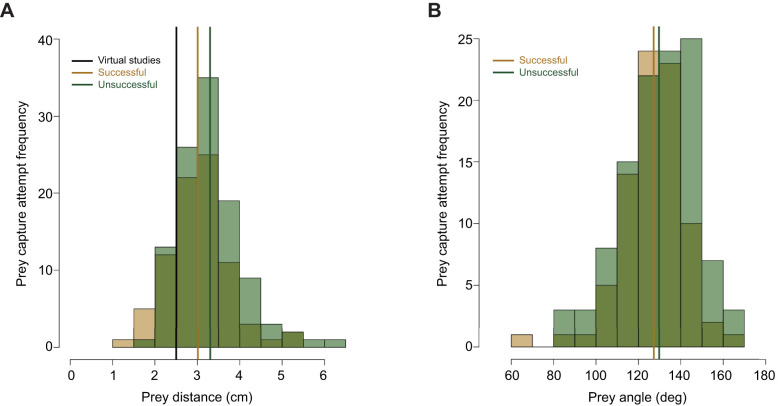
**Histograms of prey position in successful (gold) and unsuccessful (green) prey capture attempts.** (A) Prey distance; (B) prey angle. Solid black line represents the preferred prey distance in virtual studies (Nityananda et al., 2016a), gold line the mean from successful attempts, and green line the mean from unsuccessful attempts.

The praying mantises in this study initiated prey capture attempts at a mean distance of 3.18±0.054 cm (mean±s.e.m.), with a range of 1.103 to 6.218 cm (Fig. 2A). Successful attempts were closer to predictions from virtual studies, with a mean of 3.012 cm, compared with unsuccessful attempts that were significantly farther away (mean 3.31 cm; ANOVA, *F*_1,190_=7.368, *P*=0.00725; Fig. 2A). Prey angle averaged 128.71±1.16 deg (s.e.m.), with a range of 68.55–168.17 deg. Prey angle did not differ significantly between successful (mean=127.26 deg) and unsuccessful (mean=129.78 deg) attempts (*F*_1,190_=1.156, *P*=0.284).

Results from the PCA and broken stick model resulted in four PC axes that explained more variation than random and 64.7% of the total variation (Table 1). PC1 (22.85%) showed that almost all the kinematic variables loaded positively on the axis along with prey distance (Table 1). This represents prey capture attempts when prey are farther away, with bigger angles of each joint, and higher angular velocities of each joint. Both sweep time and approach time loaded negatively on PC1, representing slower prey capture attempts. PC2 (18.71%) was influenced by prey angle relative to the predator, which resulted in strikes with greater lateral displacement of the forelegs, increased body displacement and velocity, and longer sweep times to reach the prey. However, all angular velocities of each joint were lower for prey at greater angles. These first two PC axes, which together explained 41.6% of the variation in the attempts, demonstrate differences when prey were farther away (linear position of the prey relative to the eye of the mantis) or at higher angles (angle of the prey in relation to the eye of the mantis). The other two significant PC axes are summarized in Table 1. Together, they explain an additional 23.15% of the variation and highlight the diversity of movements mantises may make when attempting to capture prey. For example, PC3 (12.58%) is indicative of attempts with a prey at a high angle that are far from the mantis, which results in greater angles of the coxa and femur and more body displacement, but less lateral displacement of the forelegs.

**Table 1.**
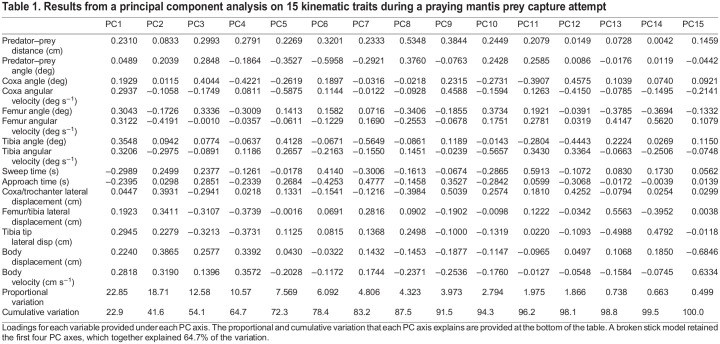
Results from a principal component analysis on 15 kinematic traits during a praying mantis prey capture attempt

**Fig. 3.**
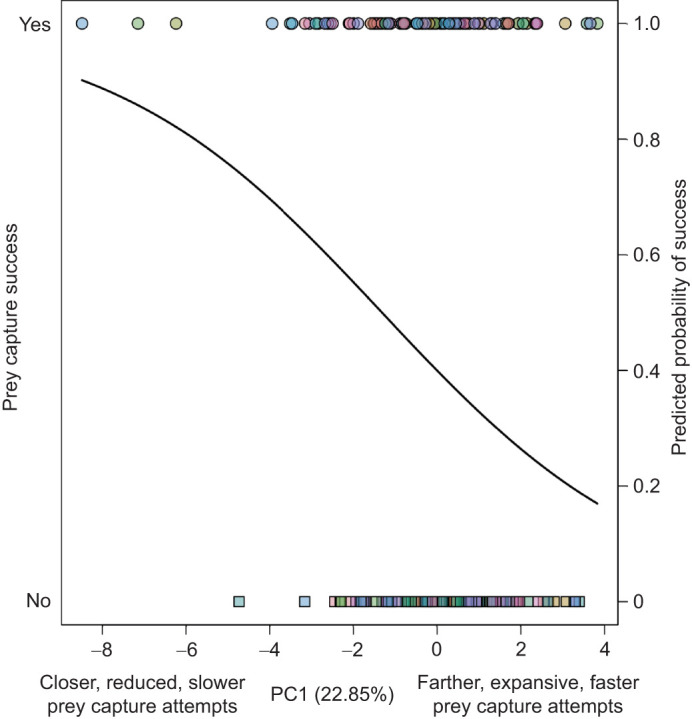
**Predicted probability of success (solid black line) based on a logistic regression model with PC1–4 predicting success.** Points represent each attempt, colors represent individuals, circles represent attempts resulting in successful capture, and squares represent unsuccessful captures.

**Fig. 4.**
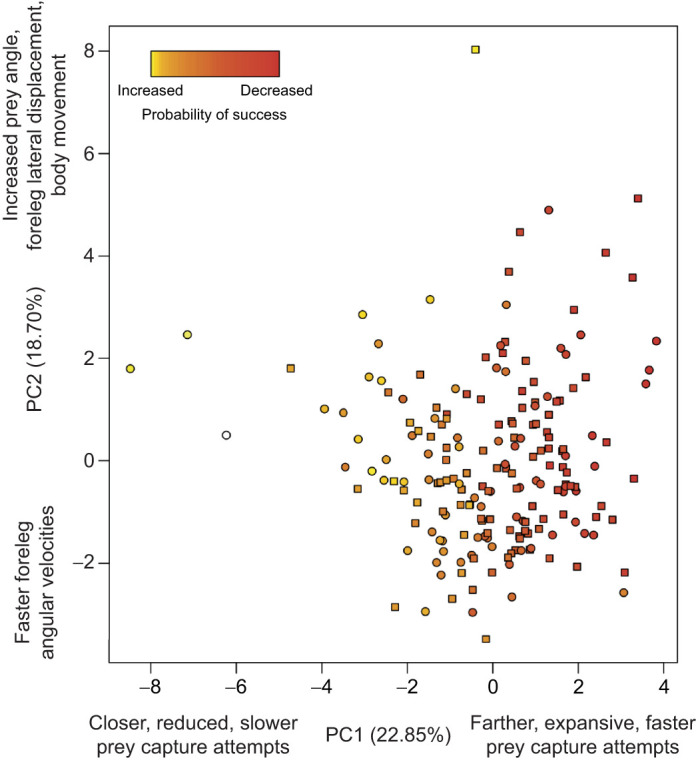
**PC1 versus PC2, points represent individuals prey capture attempts (squares, unsuccessful; circles, successful).** Colors represent predicted probability of success of the attempt based on the logistic regression model of PC1–4 predicting success. Hot colors (white) are the attempts that had the highest predicted probability of success, red represents attempts with the lowest probability of success.

Out of the strikes recorded, 110 were unsuccessful attempts, where the predator did not capture the prey and bring it to its mandibles for ingestion, and 82 were successful. Unsuccessful and successful attempts resulted in similar patterns of kinematics (Fig. 1). Using logistic regression with the first four PC axes as predictors for success, only PC1 had a significant negative effect on prey capture success (β=−0.2168, *P*=0.0103; Fig. 3). This result suggests that failed attempts are determined by prey that are farther away with attempts that are faster and exhibit greater expansion of the forelegs (Fig. 4). None of the other PC axes (PC2–4) had a significant effect on capture success (*P*>0.05).

## DISCUSSION

The ability of a predator to modulate their prey capture kinematics in response to variation in visual cues suggests that the alterations in movement may increase chances of successfully capturing the prey. Previous studies have shown that praying mantises can detect distances from 2.5 to 10 cm and that they exhibit the greatest probability of initiating a prey capture attempt at 2.5 cm (Nityananda et al., 2016a; Rosner et al., 2020). Our results show that mantises attempt to capture prey at a variety of distances, with most occurring slightly above the range of depth perception observed in studies with virtual prey, where the prey was shown on a screen in 3D space (Fig. 2). Our results also show that under laboratory conditions, which should be more favorable for predators in terms of capturing prey, predators failed more when the prey where farther away and the mantises extended their arms greater and faster. This suggests that mantises may prefer to initiate prey capture attempts at closer distances as there is an increased probability of capture success (Figs 3, 4). However, the PC axis predicting success only explained ∼22% of the variation of all strikes, with the other 78% of the variation not related to success. These results from 3D kinematics highlight the ranges of motion praying mantises exhibit when attempting to capture a prey, as they are moving two forelegs, each with three segments, in addition to their body to attempt to capture prey (Fig. S2 and Movies 1, 2). This greater range of motion may give mantises a greater target area, allow them to try and capture a greater scope of prey items, or may be modulated in response to variation in other aspects of the prey (e.g. prey size, speed, movement, etc.).

We found that adult female *S. lineola* attempt to capture prey on average at greater distances (∼3.2 cm) than what has been previously shown with virtual prey items (2.5 cm), but within the range of depth perception (2.5–10 cm) shown in neurological studies (Nityananda et al., 2016a; Rosner et al., 2020). The higher average prey distance in our study compared with virtual studies may be due to the virtual studies being limited by the distances that were presented (Nityananda et al., 2016a), which did not include the preferred distance found in this study. Therefore, it is possible that mantises presented with a virtual prey item would prefer to initiate prey capture attempts at the distances shown here, if presented with them.

**Fig. 5.**
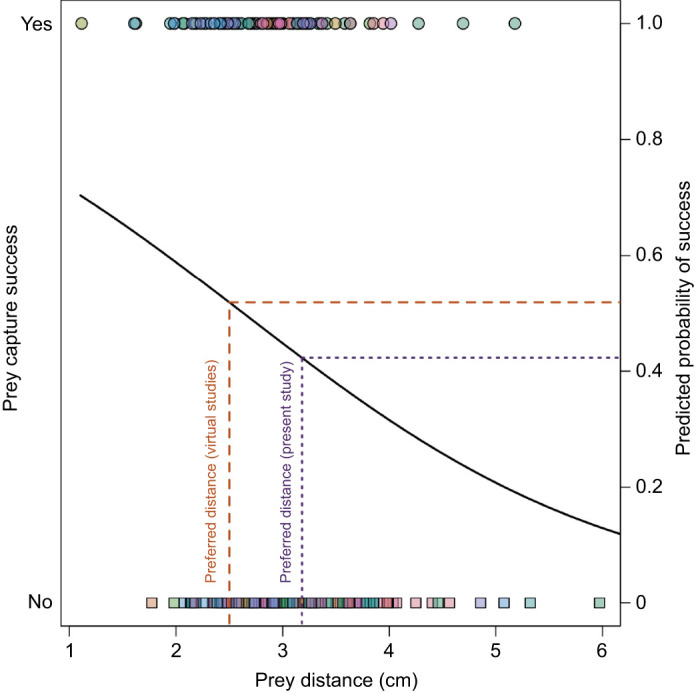
**Prey capture success as a function of prey distance alone.** As prey distance increased, prey capture success decreased. Solid black line represents the predicted probability of success based on a logistic regression model with only prey distance as a predictor. Dashed orange line represents the predicted probability of success when prey are at the preferred distance shown in virtual studies (Nityananda et al., 2016a), dashed purple line represents the predicted probability of success when prey are at the average prey distance from the present study.

Another aspect that may contribute to the difference in preferred prey capture distance is the incorporation of body lunge. The mantises in our study were unrestrained and allowed to use body lunge to close the predator–prey gap (Copeland and Carlson, 1979; Corrette, 1990; Oufiero, 2022; Oufiero et al., 2016). This suggests that mantises may prefer to initiate prey capture attempts when prey are farther away when they can incorporate body lunge. Mantises can use a substantial amount of body movement to close the predator–prey gap (mean±s.e.m. body displacement=1.60±0.058 cm), and both prey distance and body displacement load in the same direction on PC1. Therefore, when mantises are attempting to capture prey that are farther away, they use more displacement of their body to close the predator–prey gap (Copeland and Carlson, 1979; Maldonado et al., 1967). The additional distance due to movement of the body may allow mantises to remain cryptic longer or increase their chances of capturing prey as their reach is limited by their foreleg lengths (Oufiero, 2020). To explore the effects of prey distance alone on success, we examined a logistic regression model with just prey distance predicting success. Similar to the effects of PC1, prey distance had a significant, negative effect on prey capture success (β=−0.5656, *P*=0.0091). Examining the success of predators initiating an attempt at 2.5 cm (virtual preferred distance) shows they have a 51% chance of capturing the prey, whereas that probability decreases to ∼42% when attempting to capture prey based on the average distance (∼3.2 cm) seen in this study (Fig. 5). This suggests that body displacement could help increase chances of success for closer prey, but to fully determine the effects of lunge on success, studies could measure success of prey capture with and without the use of body movement to fully determine its importance.

A number of factors may contribute to a predator being successful in its attempt to capture prey, including prey distance, behavior of the prey, as well as aspects of the predator's attempt. A recent simulation study has suggested that the success of the predator may be due to informational constraints, limitations imposed by the time needed for sensory feedback, rather than biomechanical variation (Martin et al., 2022). If informational constraints are a main determinant of success in a prey capture attempt, then traits such as the timing of the attempt may be more important. This may relate to hypotheses posed by others that suggested a trade-off between speed and accuracy in decision making, including prey capture attempts (Chittka et al., 2009). Furthermore, timing during the encounter may relate to the distance of the prey, as farther prey may require faster motions to ensure capture, but not leave time for sensory-motor processing.

Some support for informational constraints has been found in a variety of taxa (Drost, 1987; Fernandez et al., 2017; Ferry-Graham et al., 2001a,b; Kane and Higham, 2014; Ladau, 2003; Maciver et al., 2001; Maie et al., 2014; Nishikawa and Roth, 1991). For example, unsuccessful prey capture in rattlesnakes is due in part to poor timing of the predator (Higham et al., 2017); in pike cichlids (*Crenicichla* sp.), unsuccessful prey capture was shown to be due in part to the predator being too fast (Milton and Bergmann, 2023); and lastly, lionfish (*Pterois volitans*) prey capture success was partly determined by the distance of a live fish prey (Peterson and McHenry, 2022). Many of these previous studies did not incorporate the kinematics of the attempt to determine how their modulation may contribute to predation success. Those that did, found that prey distance was still one of the main determining factors (Maie et al., 2014).

Our results offer some additional support for hypotheses of a speed–accuracy trade-off and informational constraints determining prey capture success. Successful attempts tended to be slower and closer, suggesting the mantises had more time to process the information and be accurate, whereas faster strikes (increased velocity of forelegs and decreased time of the attempts) were less successful and on prey farther away (Fig. 3, 4). Additional studies examining the detailed mechanisms of failed predation attempts, which can account for up to 80% of predation encounters (Abrams, 1989; Vermeij, 1982), will help to determine whether prey position, such as distance, the kinematic variation of the predators, or a combination, determines the success of predators during capture attempts.

Our study focused on prey distance, which is only one aspect of the prey mantises may be using to adjust their prey capture attempts. Depth perception is due in part to the interocular distance in mantises, which varies within species ontogenetically and among species (Kral and Poteser, 2009; Oufiero, 2020). Ontogenetically, studies have demonstrated variation in the average distance at which mantises initiated a prey capture strike, with younger individuals initiating strikes closer owing to the smaller interocular distance, but also using more lunge to close the predator–prey gap (Oufiero, 2022). Whether interspecific variation in interocular distance results in variation of preferred distance of prey capture attempts has yet to be examined.

Other studies have incorporated additional visual cues, emphasizing their potential importance. For example, Nityananda et al. (2016a) were also able to control for angular size independent of disparity cues to distance (i.e. size perceived based upon actual size of the prey and distance of the prey from the eyes) of the prey virtually, showing interactions among size, disparity-defined distance and angular size in the probability to attempt to capture the prey. However, Rossel (1991) found no direct relationship between image size and prey distance, suggesting that size constancy may not play a role in distance measurement, particularly for species that may select larger prey items such as *S. lineola*. We were unable to determine angular size of our mealbug prey item, owing to the random curvature and bending of the prey during trials, as opposed to a more consistently shaped prey (e.g. a fly). However, there was no effect of prey mass or interaction of prey mass and distance on success (*P*>0.05, results not shown). Nevertheless, incorporating additional aspects of prey size, distance and angular size might relate to the other components of prey capture attempt kinematic variation captured by the additional PC axes and prey capture success that is not related to prey distance (Table 1).

Additional studies have shown that contrast of the prey, prey movement and prey speed all can affect components and probability of a prey capture attempt in mantises (Iwasaki, 1991; Prete et al., 1993, 2013; Rossel, 1991; Rossoni and Niven, 2020; Yamawaki, 2017). Each of these characteristics may affect the motor outputs controlling the prey capture attempt to result in kinematic variation. For example, Rossoni and Niven (2020) found that as prey speed increased, both tibial extension and probability of the mantis pausing both decreased. Furthermore, success in prey capture attempts may also be related to these other visual cues, as Rossoni and Niven (2020) showed a decrease in probability of success as prey speed increased. We examined the movement of the prey during the approach phase and found it did not have a significant effect on success (*P*>0.05, results not shown). However, we did not incorporate movement prior to the attempt like other studies (Borghuis and Leonardo, 2015; Peterson and McHenry, 2022). Adding interactive effects of the prey type in studies (e.g. distance and size of prey) may help separate out the strategies mantises use to capture variable types of prey and their success in attempts.

The prey capture attempts of mantises were summarized by four PC axes that explained 64.7% of the variation and highlight the variation and relation of kinematic traits during successful and failed attempts (Table 1). Previous studies of praying mantis prey capture have examined the components independently (Copeland and Carlson, 1979; Corrette, 1990; Maldonado et al., 1967; Oufiero, 2022; Oufiero et al., 2016). Principal component analysis is one of the more common ways to summarize functional data, which is more useful in the mantis system that exhibits a range of motions during prey capture attempts, and may be applied to future studies taking a comparative approach to examine the kinematic variation of prey capture (Baliga and Mehta, 2015; Gillis and Lauder, 1995; Higham, 2007; Meyers and Herrel, 2005; Oufiero et al., 2012). Incorporating the visual aspects of the prey (prey distance and prey angle) allows for the reduction of kinematic variables with visual cues of the prey to determine both their relationship and the main axes of variation, similar to previous studies that incorporated prey distance during predation events in other taxa (Meyers and Herrel, 2005; Oufiero et al., 2012). Both prey distance and angle have been examined previously in mantises and were shown to affect aspects of successful prey capture attempts (Oufiero, 2022; Oufiero et al., 2016). Our results suggest that mantises may be adjusting strikes depending on where the prey is (how far away or above its head) when they initiate an attempt, but only distance and the associated kinematic adjustments are predicting success (Fig. 4).

Based on our results, PC1 (22.85%) describes strikes where the mantises modulate their kinematics based on prey distance in a medial plane (e.g. greater angles of each joint and more body movement). Similar results have been found in cuttlefish, another invertebrate with stereoscopic vision, where an increase in virtual prey distance resulted in an increase in tentacle extension (Feord et al., 2020). PC2 (18.71%) described attempts in which the mantises increased the lateral displacement of their forelegs in relation to prey angle. This is the first study to incorporate 3D kinematics of the praying mantis prey capture and results from PC2, lateral displacement, highlights an axis that has been missing from previous studies (Cleal and Prete, 1996; Copeland and Carlson, 1979; Corrette, 1990; Maldonado et al., 1967; Oufiero, 2022; Oufiero et al., 2016; Rossoni and Niven, 2020; Yoshida et al., 2023). The ranges of motion in the medial and lateral plane provide mantises with a greater scope in which they can attempt to capture prey. The different visual cues of the prey may represent different visual stimuli that may be used independently to modulate the prey capture attempt. Adding other components of the prey, such as size, speed, contrast, etc., may help explain the other variation seen in prey capture attempts (PC2–4; Table 1) and further highlight the flexibility of the rapid prey capture attempts.

Praying mantises have become models for stereoscopic vision as they are the only arthropod currently known to possess this trait (Iwasaki, 1991; Nityananda et al., 2016a,b, 2019; Rossel, 1980, 1983, 1991; Yamawaki, 2017). Recent results have begun to link aspects of the visual cues of the prey to the variation in their prey capture strikes beyond eliciting a response (Oufiero, 2022; Oufiero et al., 2016; Rossoni and Niven, 2020). Incorporating 3D kinematics with more detailed control of prey visual cues, along with successful and failed attempts, will help provide more information on how praying mantises are using visual information to modulate the kinematics of their forelegs to successfully capture prey. The flexibility in their prey capture attempts suggest that aspects may be under control from internal models (e.g. feedback or feedforward mechanisms), but more research is warranted to understand the sensory-motor processing (Perry and Chittka, 2019; Webb, 2004; Yamawaki, 2017). For example, although we did not control aspects of the prey or record orientation behaviors prior to the attempts, future studies could, which may provide more information on the neural processes (Borghuis and Leonardo, 2015; Peterson and McHenry, 2022). This system may serve as a model to understand the rapid control of complex motor patterns as well as sensory motor integration during a behavior that has direct ties to the fitness of the organism.

## Supplementary Material

10.1242/jexbio.247311_sup1 Supplementary information
