# Supplementary material for: Modulation of prey capture kinematics in relation to prey distance helps predict success
Source: J Exp Biol. 2024 Jun 12;227(11):jeb247311. doi: 10.1242/jeb.247311 (PMC11213525; doi:10.1242/jeb.247311)
Supplement: Supplementary information [file jexbio-227-247311-s1.pdf]

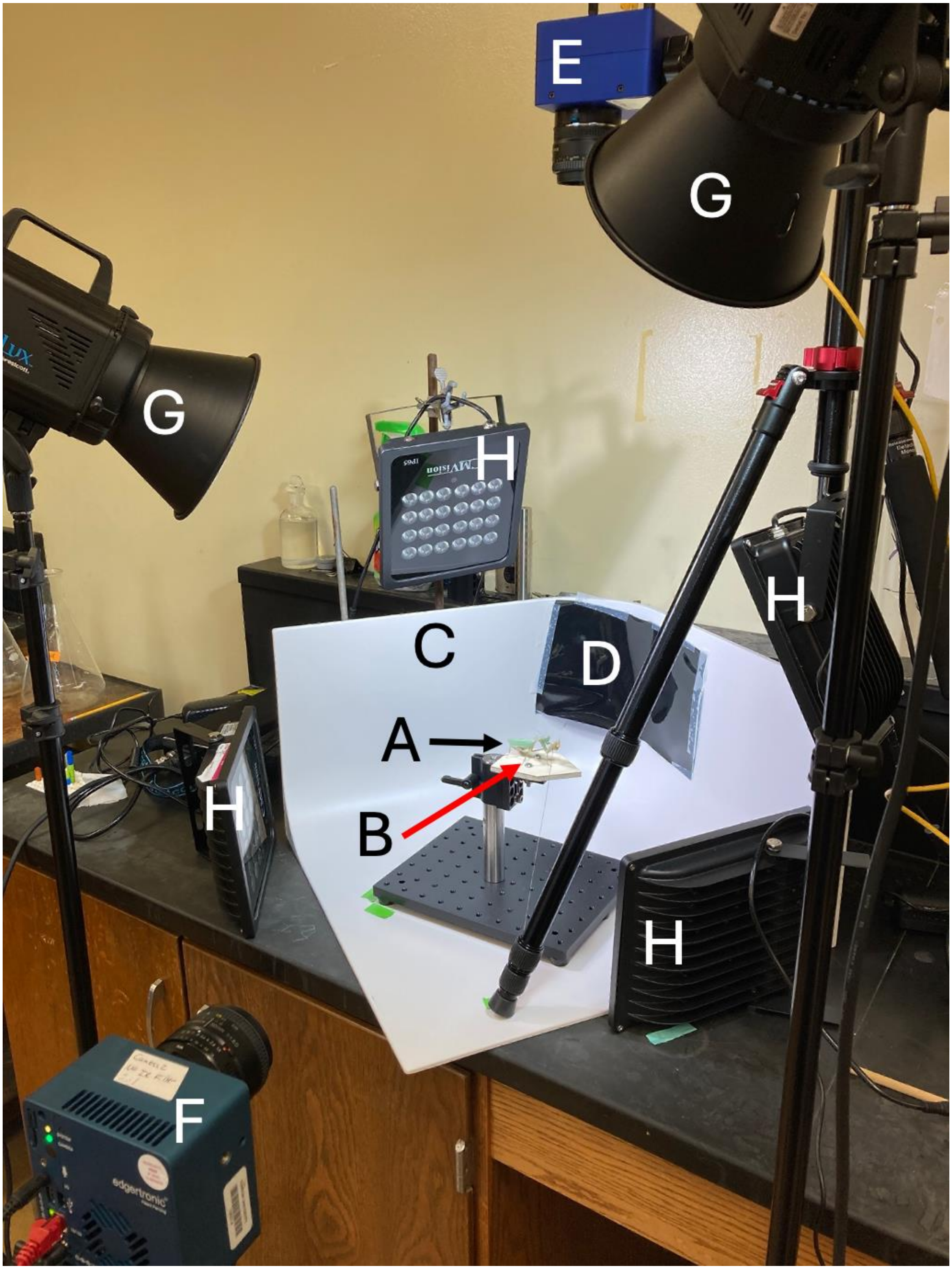


Journal of Experimental Biology • Supplementary information

**Fig. S1.** Experimental setup. Each mantis (A) was placed on the wooden platform (B) right side up in a photostudio (C). Black aquarium plastic (D) was placed behind the mantis to enhance contrast. A mealworm prey was introduced on a metal wire and moved manually in a random manner to elicit prey capture attempts. Attempts were recorded with 2 Edgertronic SC1 high-speed cameras synced at 1000 Hz, one filming dorsally (E) and one laterally (F). The filming area was illuminated with Westcott SKYLUX 1000 Watt LED Lights (G) and 4 CM Vision IR (850nm) lights (H). Some individuals were also on a plastic 32oz lid (not pictured), which is the lid of their housing container as it eased movement to the platform and reduced stress.

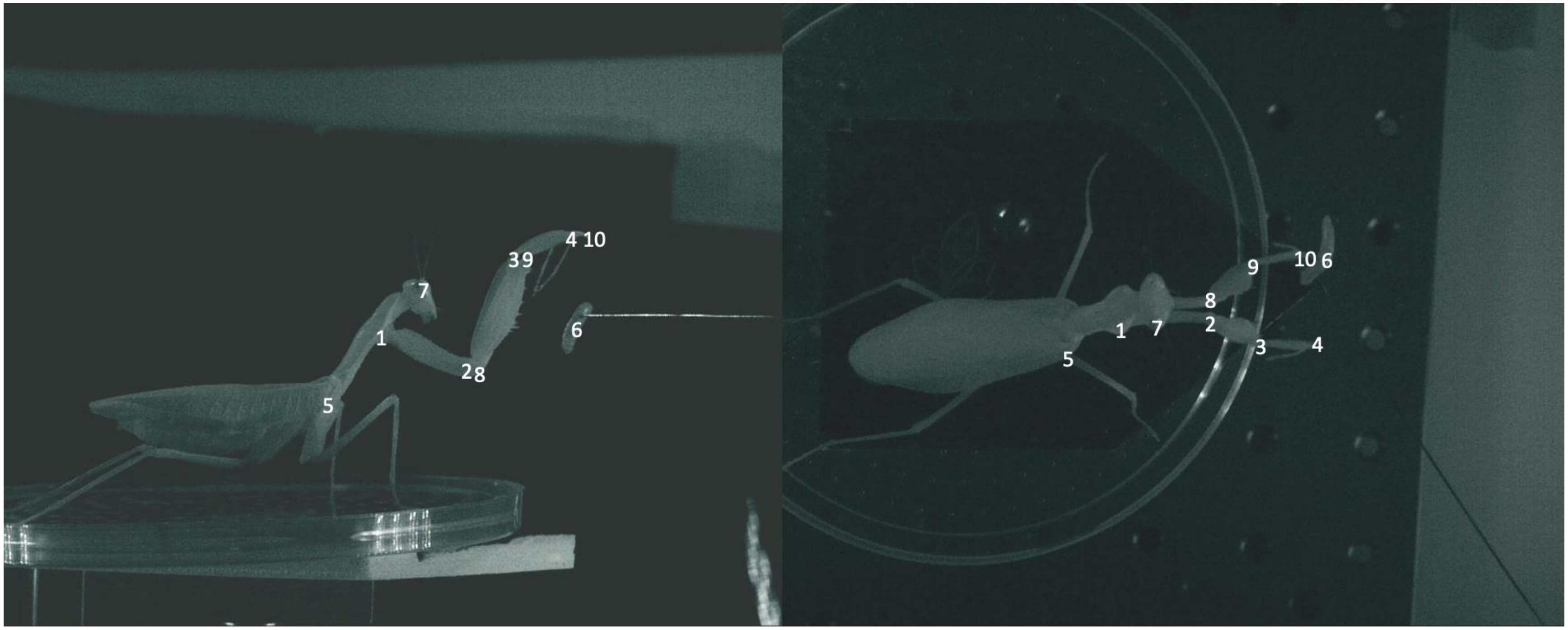


1. Front coxa-prothorax
2. Front trochanter/femur
3. Front tibia-femur
4. Front tip of tibia
5. Front Mid leg mesothorax
6. Prey
7. Front Eye
8. Back Trochanter/femur
9. Back tibia-femur
10. Back tip of tibia

**Fig. S2.** Points digitized on each view for each prey capture attempt.

Journal of Experimental Biology • Supplementary information

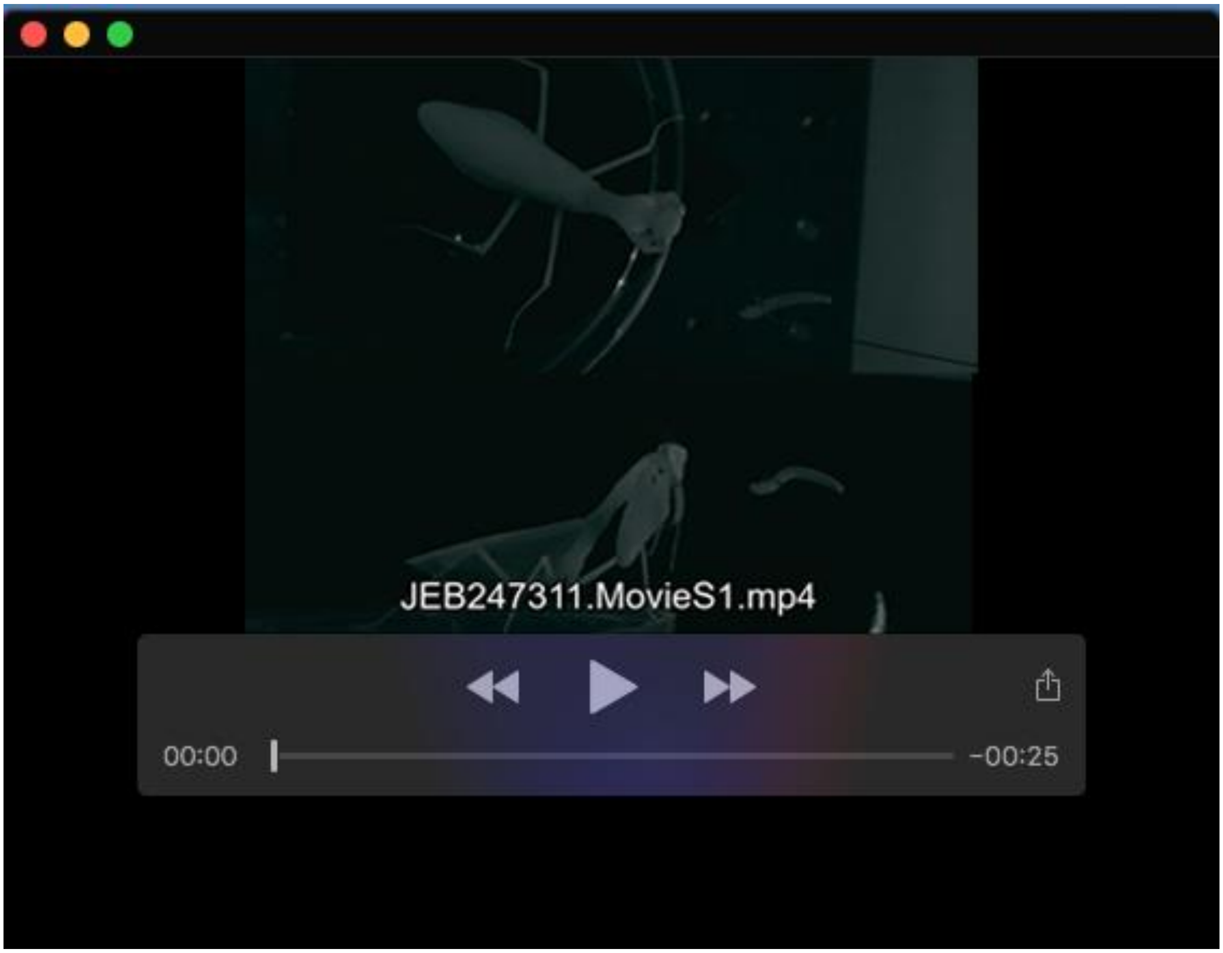


**Movie 1.** Sample video of a successful prey capture attempt. Videos captured with two synced Edgertronic SC1 cameras (Sanstreak Corporation, San Jose, CA) within 1 μs at 1000Hz frame rate. Videos played back at 30 Hz. Successful attempts were categorized as the mantis successfully capturing the prey between the femoral and tibial spines and bringing it back to its mandibles for ingestion. This video represents the same individual and the same trial day as Video 2, highlighting how successful and unsuccessful attempts could be captured on the same individual and day.

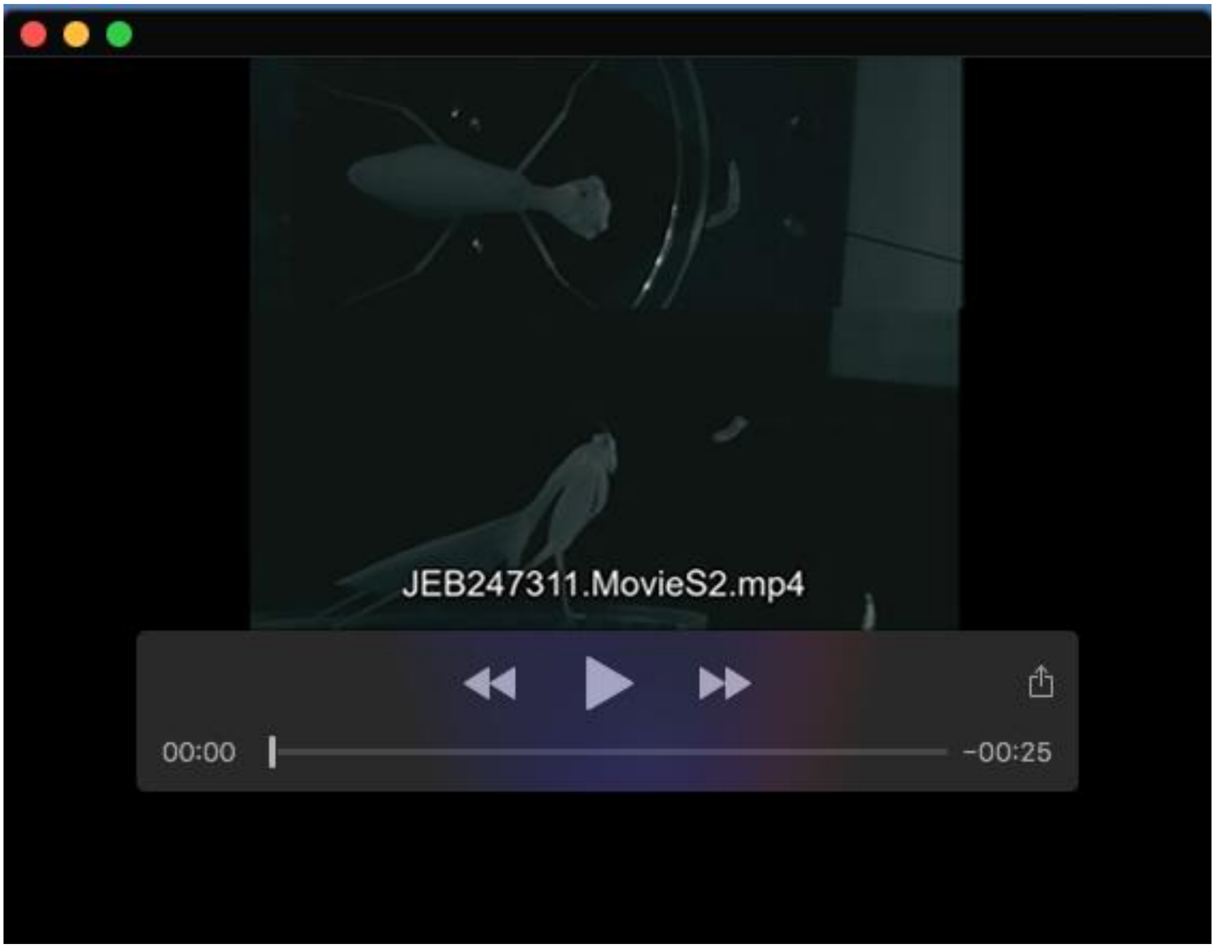


**Movie 2.** Sample video of a unsuccessful (failed) prey capture attempt. Videos captured with two synced Edgertronic SC1 cameras (Sanstreak Corporation, San Jose, CA) within 1 µs at 1000Hz frame rate. Videos played back at 30 Hz. Unsuccessful attempts were categorized as the mantis not capturing the prey between the femoral and tibial spines and bringing it back to its mandibles for ingestion. This video represents the same individual and the same trial day as Video 1, highlighting how successful and unsuccessful attempts could be captured on the same individual and day.

Journal of Experimental Biology • Supplementary information
